# Is race or ethnicity associated with under‐utilization of statins among women in the United States: The study of women's health across the nation

**DOI:** 10.1002/clc.23448

**Published:** 2020-08-30

**Authors:** Elizabeth A. Jackson, Kristine Ruppert, Carol A. Derby, Yinjuan Lian, Claudia U. Chae, Rasa Kazlauskaite, Genevieve Neal‐Perry, Samar R. El Khoudary, Siobán D. Harlow, Daniel H. Solomon

**Affiliations:** ^1^ Division of Cardiovascular Disease, Department of Internal Medicine University of Alabama at Birmingham Birmingham Alabama USA; ^2^ Department of Epidemiology Graduate School of Public Health, University of Pittsburgh Pittsburgh Pennsylvania USA; ^3^ The Saul R. Korey Department of Neurology, and Department of Epidemiology and Population Health Albert Einstein College of Medicine Bronx New York USA; ^4^ Division of Cardiology Massachusetts General Hospital Boston Massachusetts USA; ^5^ Department of Medicine Rush University Medical Center chicago Illinois USA; ^6^ Department of Obstetrics and Gynecology University of North Carolina Chapel Hill North Carolina USA; ^7^ Department of Epidemiology, School of Public Health University of Michigan Ann Arbor Michigan USA; ^8^ Division of Rheumatology, Division of Pharmacoepidemiology Brigham and Women's Hospital, Harvard Medical School Boston Massachusetts USA

**Keywords:** cardiovascular prevention, race/ethnicity, statin therapy, women

## Abstract

**Background:**

Rates of statin use among minority women are unclear.

**Hypothesis:**

We hypothesized that statin use would vary by race/ethnicity with lower rates among minority women compared with Whites.

**Methods:**

Data from the study of women's health across the nation, a multiethnic cohort of women collected between 2009 to 2011 were used to examine reported statin use by race/ethnicity and risk profile. Multivariable logistic modeling was performed to estimate the odds ratio (OR) of statin treatment.

**Results:**

Of the 2399 women included, 234 had a diagnosis of atherosclerotic disease (ASCVD), 254 were diabetic (without ASCVD), 163 had an LDL ≥190 mg/dL, and 151 had a 10 year ASCVD pooled risk score ≥7.5%. Statins were used by 49.6% of women with CVD; 59.8% of women with diabetes without known ASCVD; 42.3% of women with an LDL ≥190 mg/dL; and 19.9% of women with an ASCVD risk ≥7.5%. Rates of statin use were 43.8% for women with ≥ two prior ASCVD events and 69.4% for women with ≥ one prior ASCVD event plus multiple high‐risk conditions. Among women eligible for statins, Black women had a significantly reduced adjusted odds of being on a statin (OR 0.53, 95% confidence interval [CI] 0.36‐0.78) compared with White women.

**Conclusions:**

In this cohort of multiethnic women, rates of statin use among women who would benefit were low, with Black women having lower odds of statin use than White women.

## 1 INTRODUCTION

For women, in particular, minority women, CVD is a leading cause of morbidity and mortality in the United States (US).^1^, ^2^, ^3^ For over two decades, guidelines and associated clinical trials have recommended statin therapy for women with a history of CVD or CVD risk factors.^4^, ^5^, ^6^, ^7^ In 2013, the American Heart Association (AHA) and American College of Cardiology (ACC) guidelines emphasized statins use for four primary groups.^7^ These groups include those with (a) atherosclerotic cardiovascular disease (ASCVD); (b) diabetes, age 40 to 75 years; (c) an LDL cholesterol ≥190 mg/dL; or (d) a 10 year ASCVD risk score ≥7.5%.^8^, ^9^

Prior studies report women are less likely to receive evidence‐based therapies including statins compared to men.^10^, ^11^, ^12^, ^13^ However, data on rates of statin use among minority women for these statin eligible groups remains less studied. The study of women's health across the nation (SWAN) is a multiethnic prospective cohort study of midlife women, which includes data on CVD risk factors and medication use. We sought to describe patterns of statin use among women by race/ethnicity, using data from the 12th SWAN study visit in 2009 to 2011. We hypothesized that statin use would be lower for Black and Hispanic women with a history of ASCVD, diabetes, or elevated LDL cholesterol compared to White women. We also estimated the number of women by race/ethnicity, who would have been advised to initiate statin therapy secondary to having an ASCVD risk of 7.5% or higher.

## 2 METHODS

SWAN is a community‐based longitudinal study that includes a multiethnic population of women transitioning through menopause. The study design has been described in detail elsewhere.^14^ A total of 3302 women were enrolled in seven sites within the US, used various sampling frameworks and recruitment strategies to enroll representative groups of women from the surrounding communities. All sites recruited non‐Hispanic Whites. Specific racial/ethnic groups were recruited from different sites. Black women were enrolled in the Boston, Massachusetts, Chicago, Illinois, Pittsburgh, Pennsylvania, and southeast Michigan sites. Chinese and Japanese women were enrolled at the Oakland, California, and Los Angeles, California, sites. Hispanic women were enrolled at the Hudson County, New Jersey site.

All women enrolled were age 42 to 52 years upon entry and were perimenopausal. Women were ineligible if they reported no menses in the prior 3 months before enrollment. Women who had a hysterectomy or bilateral oophorectomy were also excluded, as were women taking oral contraceptives or hormonal therapy in the prior 3 months. Additional exclusion criteria, including a history of CVD prior to enrollment. All participants provided written informed consent prior to enrollment. Each site obtained approval for the study from their respective Institutional Review Board.

To be eligible for this analysis, women had to attend visit 12 and have no missing data for ASCVD risk scoring. The women were categorized as statin eligible if they fit into one of the four groups including: (a) a history of ASCVD diagnosed during follow‐up; (b) a diagnosis of diabetes and age 40 to 75 years; (c) an LDL‐cholesterol ≥190 mg/dL; or (d) a 10 year ASCVD risk score ≥ 7.5%. To calculate the 10 years ASCVD score, we used the 2013 ACC/AHA guidelines for cardiovascular risk assessment.^8^, ^9^ Each woman could contribute to only one group; thus, a woman who had a diagnosis of CVD and had diabetes were grouped with those diagnosed with CVD. The ASCVD risk score was only calculated for women who were not included in the other three groups.

The 2013 ACC/AHA 10 year ASCVD risk score included blood pressure, a diagnosis of hypertension (HTN), a diagnosis of diabetes (defined as a history of self‐report, fasting blood glucose >126 or use of antiglycemic medications), current smoking, total cholesterol, and high density lipoprotein cholesterol (HDL‐C)levels, in addition to race and age.^8^, ^9^ Systolic and diastolic blood pressure (DBP) was measured using a standardized protocol, with two resting measurements averaged. HTN was defined as systolic blood pressure (SBP) ≥140 mmHg, diastolic blood pressure ≥90 mmHg, or current use of antihypertensive medications (queried at each annual visit). Smoking status at visit 12 was categorized as never/past/current. Body mass index (BMI) (kg/m^2^) was calculated using the height and weight collected at visit 12. All lipid, lipoprotein, and apolipoprotein fractions were analyzed on ethylene diamine tetraacetic acid (EDTA)‐treated plasma.^15^, ^16^ Total cholesterol and triglycerides were analyzed by enzymatic methods on a Hitachi 747 analyzer (Boehringer Mannheim Diagnostics, Indianapolis, Indiana) as previously described.^15^ HDL‐C was isolated using heparin‐2 M manganese chloride.^16^ When triglycerides were less than 400 mg/dL, and the calculated LDL result was obtained by using the Friedewald formula (LDL = total cholesterol ‐ HDL ‐ triglycerides /five).

Women were considered at high‐risk for future ASCVD events if they had two or more ASCVD events prior to visit 12 or had one ASCVD event and two or more high‐risk conditions including age ≥ 65 years, and LDL ≥100 mg/dL, diabetes mellitus, HTN, current smoking.^17^ ASCVD events were self‐report. We did not have enough information to consider chronic kidney disease, congestive heart failure, or the guidelines' definition of persistently elevated LDL on maximally tolerated statin therapy and ezetimibe.

Participants were asked about medications at each study visit, and responses were verified by inspection of medication bottles. If the participant forgot to bring medication containers to the study visit, a review of medication lists was performed. Dosage information was not consistently provided and thus was not used for these analyses.

Demographic factors, including age, race/ethnicity, and socioeconomic (SES) characteristics (highest education level attained, income), were collected at baseline. Income was categorized as (< 20 K, 20‐<35 K, 35‐<50 K, 50‐<75 K, and 75 K or more) and financial strain was assessed based on asking each participant was asked “how hard is it to pay for basics” with possible responses ranging from “very hard” to “somewhat hard” to “not hard at all.” Medical history was collected via self‐report at annual visits. ASCVD (including myocardial infarction, percutaneous coronary intervention or coronary artery bypass grafting, peripheral arterial disease, and stroke or transient ischemic attack), diabetes, and renal disease were asked at each visit. A family history of premature coronary artery disease (CAD) was defined as CAD in first‐degree relatives (male ≤55 years or female ≤65 years).

Menopausal status was defined as pre, peri (early, late), and postmenopausal according to standard definitions.^8^ A woman who reported a menstrual period within the past 3 months with no change in regularity was considered premenopausal. Early perimenopause was defined as a menses within the past 3 months with a change in regularity. Women who reported no menses for ≥ three, but at least one period within the last 12 months was considered late perimenopausal. No menses for ≥ 12 consecutive months was considered as postmenopause if no other reason for the amenorrhea was evident. For these analyses, we classified menopausal status as a surgical postmenopause if they had a hysterectomy with bilateral salpingo‐oophorectomy. Natural post menopause is defined as women who have gone through menopause naturally. Early and late perimenopause were combined, and no women were premenopausal at V12. A woman's menopausal status was categorized as unknown if she reported the use of hormonal therapy or information was missing.

We described the participant characteristics in each racial/ethnic group using descriptive statistics (mean, median, and range). Continuous variables were analyzed using Student's *t*test or ANOVA. Wilcoxon Rank Sum and Kruskal‐Wallis tests were used when the data were not normally distributed. Categorical variables were analyzed using chi‐square tests or exact tests where appropriate. Logistic regression was used to examine racial/ethnic differences among women eligible for statin use. Since income contains a large number of missing values, we decided to use financial strain in the models. We chose variables for the multivariable analyses a priori based on possible confounders and important covariates. Model 1 was adjusted for race, age, BMI, education, difficulty paying for basics, hormone therapy use, and menopausal status. Model 2 included study site, smoking status, family history of premature CAD, and diagnosis of HTN, in addition to the variables listed above. Results are presented as odds ratios and 95% confidence intervals.

After grouping participants into the four statin eligible groups, we examined if there were racial/ethnic differences in statin use within each group using the same methodology as above. Since there were not enough Asian or Hispanic women in the group with the 10 year ASCVD risk ≥7.5%, we could only examine the differences between the White and Black women. All analyses were performed using SAS software 9.3 (Cary, North Carolina).

## 3 RESULTS

**FIGURE 1:**
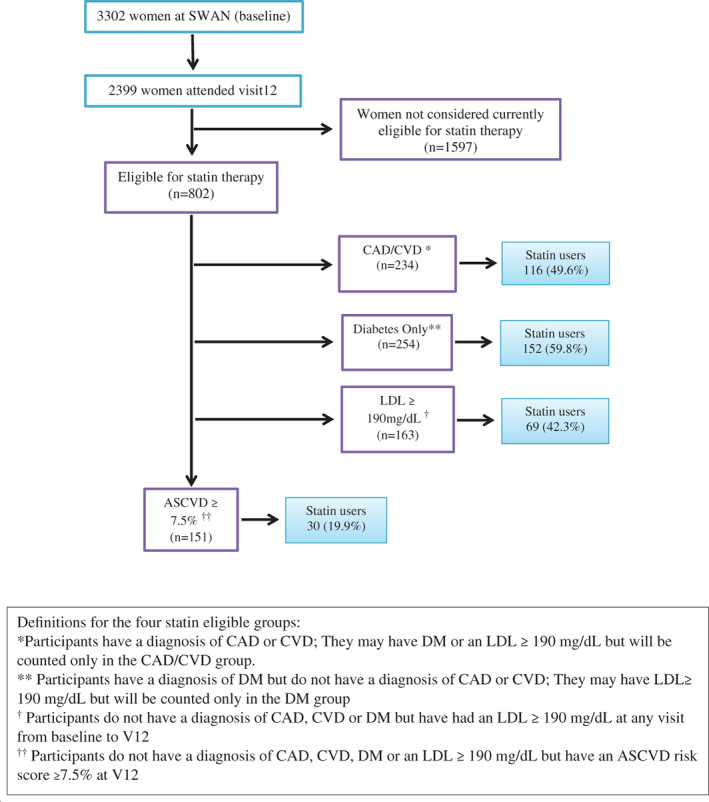
SWAN participants by statin eligibility: consort diagram. SWAN, study of women's health across the nation

Of the women who attended visit 12 (n = 2399), a total of 802 (33%) women met criteria for one of the four groups recommended for statin therapy including 234 (29.2%) women reporting an ASCVD event, 254 (31.7%) with diabetes (without ASCVD), 163 (20.3%) with a history of an LDL‐cholesterol level ≥ 190 mg/dL, and 151 (18.8%) with a 10 year ASCVD risk of 7.5% or higher. (Figure 1). A total of 1597 women either had a 10 year ASCVD risk score <7.5% or were missing data to calculate the score and were excluded from the analyses.

Women who met eligibility criteria for statins were slightly older and more likely to be Black or Hispanic compared to women who did not meet the criteria (Table 1). SES factors also differed between the two groups, with more women who were statins eligible reporting lower levels of education and income and higher rates of difficulty paying for basics. As expected, CVD risk factors were more prevalent among women who met the criteria for statins as compared to women who did not.

**TABLE 1:** Characteristics of women Who attended Visit 12 by statin eligibility status

Characteristic, n (%)	Total N = 2399	Eligible for statin therapy N = 802	Not eligible for statin therapy N = 1597	*P*‐value
Age, years, mean (SD)	60.2 (2.7)	60.6 (2.9)	60.0 (2.6)	<.0001
Race/Ethnicity				<.0001
White	1159 (48.3)	310 (38.7)	849 (53.2)	
Black	662 (27.6)	351 (43.8)	311 (19.5)	
Hispanic	131 (5.5)	56 (7.0)	75 (4.7)	
Asian	447 (18.6)	85 (10.6)	362 (22.7)	
Education				<.0001
≤ High School	507 (21.3)	219 (27.6)	288 (18.2)	
> High School	773 (32.5)	306 (38.5)	467 (29.5)	
College	511 (21.5)	123 (15.5)	388 (24.5)	
Post	588 (24.7)	146 (18.4)	442 (27.9)	
Income				<.0001
Less than $20 000	229 (11.2)	138 (20.0)	91 (6.7)	
$ 20 000‐35 000	249 (12.1)	103 (15.0)	146 (10.7)	
$ 35 000‐50 000	278 (13.6)	110 (16.0)	168 (12.3)	
$ 50 000‐75 000	408 (19.9)	135 (19.6)	273 (20.0)	
$ 75 000 or more	887 (43.3)	203 (29.5)	684 (50.2)	
How hard to pay basics				<.0001
Very hard	138 (6.4)	80 (10.8)	58 (4.0)	
Somewhat hard	534 (24.6)	235 (31.8)	299 (20.8)	
Not hard at all	1502 (69.1)	423 (57.3)	1079 (75.1)	
Hypertension^b^	1093 (50.5)	565 (75.0)	528 (37.4)	<.0001
Family history of CAD^a^	1457 (63.9)	526 (69.9)	931 (60.9)	<.0001
Current Smoker	228 (11.0)	144 (20.6)	84 (6.1)	<.0001
BMI, (kg/m^2^), mean (SD)	29.5 (7.4)	32.5 (7.8)	28.0 (6.7)	<.0001
Waist circumference, cm, mean (SD)	91.6 (16.4)	99.0 (16.7)	87.8 (14.9)	<.0001
Waist hip ratio, mean (SD)	0.8 (0.1)	0.9 (0.1)	0.8 (0.1)	<.0001
Systolic blood pressure (mmHg), mean (SD)	122.0 (17.1)	129.9 (19.3)	117.9 (14.2)	<.0001
Diastolic blood pressure (mmHg), mean (SD)	74.5 (10.3)	76.3 (11.2)	73.6 (9.6)	<.0001
Total cholesterol (mg/dl), mean (SD)	204.8 (38.4)	201.2 (46.9)	206.7 (33.0)	<.0001
High density lipoprotein (mg/dl), mean (SD)	62.2 (16.6)	56.4 (15.2)	65.2 (16.5)	<.0001
Low density lipoprotein (mg/dl), mean (SD)	119.4 (32.5)	119.0 (40.8)	119.6 (27.1)	.01
Triglycerides (mg/dl), mean (SD)	117.5 (62.9)	133.9 (73.3)	108.9 (54.7)	<.0001
Menopausal status				.04
Surgical post	143 (6.0)	61 (7.6)	82 (5.1)	
Natural post	2116 (88.2)	703 (87.7)	1413 (88.5)	
Early/late perimenopause	65 (2.7)	17 (2.1)	49 (3.1)	
Unknown^c^	74 (3.1)	21(2.6)	53 (3.3)	
Years since FMP, mean (SD)	8.7 (3.2)	8.7 (3.1)	8.5 (3.0)	<.0001
Hormone users				.98
Current	173 (7.2)	51 (6.4)	122 (7.6)	
Past	853 (35.6)	288 (35.9)	565 (35.4)	
Study Site				<.0001
Michigan	406 (16.9)	244 (27.9)	182 (11.4)	
MGH	355 (14.8)	123 (15.3)	232 (14.5)	
Chicago	333 (13.9)	102 (12.7)	231 (14.5)	
UC Davis	371 (15.5)	69 (8.6)	302 (18.9)	
UCLA	409 (17.1)	81 (10.1)	328 (20.5)	
New Jersey	204 (8.5)	71 (8.6)	133 (8.3)	
Pittsburgh	321 (13.4)	132 (16.5)	189 (11.8)	

*Note*: P‐values are calculated using chi‐square and Student's *t* test or Wilcoxon rank sum.

^a^ Family history of CAD definition: CAD (coronary artery disease) in first‐degree relative male ≤55 years or female ≤65 years.

^b^ Hypertension definition: SBP ≥140 mmHg or DBP ≥90 mmHg or use of antihypertensive medications.

^c^ Unknown menopausal status includes women taking hormonal therapy and/or missing.

Abbreviations: BMI, body mass index; CAD, coronary artery disease; DBP, diastolic blood pressure; FMP, final menstrual period; SBP, systolic blood pressure.

Among Black, White, Hispanic, Asian women, 31.6%, 29.0%, 30.4%, and 18.8%, respectively, met criteria for statin eligibility by having a diagnosis of ASCVD. More women who were Hispanic (41.1%), or Asian (40.0%), met criteria for statin eligibility by having a diagnosis of diabetes (without ASCVD) compared to Black (29.1%) and White (30.7) women (Table 2). HTN was highly prevalent among statin eligible women within all racial/ethnic groups, with almost 90% of Black and Hispanic women carrying a diagnosis of HTN. Among women eligible for statin therapy, SES characteristics differed by race/ethnicity. Education levels were lowest among Hispanics. Income levels were lowest among Hispanics, followed by Blacks. More than half of all Blacks reported at least some difficulty paying for basics, while more than 70% of Whites and Asians reported no such difficulty.

**TABLE 2:** Characteristics of women eligible for statin therapy by race/ethnicity

Characteristic, n (%)	Total N = 802	Black N = 351	White N = 310	Hispanics N = 56	Asian N = 85	*P*‐value
Age, years, mean (SD)	60.6 (2.9)	60.1 (2.8)	60.9 (2.9)	60.9 (3.1)	61.5 (2.6)	<.0001
Statin benefit group						
History of CVD^a^	234 (29.2)	111 (31.6)	90 (29.0)	17 (30.4)	16 (18.8)	<.0001
Diabetes without CVD^b^	254 (31.7)	102 (29.1)	95 (30.7)	23 (41.1)	34 (40.0)	<.0001
Low density lipoprotein (mg/dl) ≥190 mg/dL^c^	163 (20.3)	42 (12.0)	84 (27.1)	9(16.1)	28 (32.9)	.85
ASCVD score of ≥7.5%	151 (18.8)	96 (27.4)	41 (13.2)	7 (12.5)	7 (8.2)	<.0001
Hypertension^d^	565 (75.0)	292 (86.7)	187 (64.7)	43 (86.0)	43 (55.8)	<.0001
Family history of CAD^e^	526 (70.0)	222 (68.9)	223 (75.1)	31 (63.3)	50 (59.5)	.03
Current smoker	144 (20.6)	87 (28.9)	41 (14.2)	10 (33.3)	6 (7.7)	<.0001
BMI, mean (SD)	32.5 (7.8)	34.2 (7.9)	32.1 (7.4)	34.0 (7.2)	26.1 (4.8)	<.0001
Waist circumference, mean (SD)	99.0 (16.7)	101.7 (15.8)	98.9 (16.7)	104.5 (17.2)	86.4 (11.3)	<.0001
Waist hip ratio, mean (SD)	0.9 (0.1)	0.9 (0.1)	0.9 (0.1)	0.9 (0.1)	0.9 (0.1)	.30
Systolic blood pressure (mmHg), mean (SD)	129.9 (19.3)	136.1 (19.3)	124.0 (16.8)	136.8 (19.5)	121.3 (17.5)	<.0001
Diastolic blood pressure (mmHg), mean (SD)	76.3 (11.2)	79.2 (11.2)	73.4 (10.3)	79.8 (11.5)	72.8 (11.0)	<.0001
Total cholesterol (mg/dl), mean (SD)	201.2 (46.9)	197.7 (44.4)	203.3 (48.5)	200.0 (45.7)	209.6 (54.4)	.54
High density lipoprotein (mg/dl), mean (SD)	56.4 (15.2)	56.0 (16.1)	56.4 (14.8)	53.6 (12.0)	59.8 (14.1)	.06
Low density lipoprotein (mg/dl), mean (SD)	119.0 (40.8)	119.4 (37.9)	118.8 (43.5)	116.9 (37.7)	102.9 (34.5)	.76
Triglycerides (mg/dl), mean (SD)	133.9 (73.3)	112.3 (53.9)	149.1 (82.8)	155.1 (78.0)	148.9 (81.8)	<.0001
Education						<.0001
≤High school	219 (27.6)	100 (28.8)	57 (18.5)	39 (72.2)	23 (27.1)	
>High school	306 (38.5)	161 (46.4)	106 (34.4)	11 (20.4)	28 (32.9)	
College	123 (15.5)	47 (13.5)	50 (16.2)	4 (7.4)	22 (25.9)	
Post	146 (18.4)	39 (11.2)	95 (30.8)	0	12 (14.1)	
Income						<.0001
Less than $20 000	138 (20.0)	78 (26.4)	27 (9.6)	27 (62.8)	6 (8.6)	
$20000‐35 000	103 (15.0)	58 (19.6)	35 (12.5)	4 (9.3)	6 (8.6)	
$ 35 000‐50 000	110 (16.0)	59 (19.9)	43 (15.4)	3 (7.0)	5 (7.1)	
$ 50 000‐75 000	135 (19.6)	57 (19.3)	54 (19.3)	6 (14.0)	18 (25.7)	
$75 000 or more	203 (29.5)	44 (14.9)	121 (43.2)	3 (7.0)	35 (50.0)	
How hard to pay basics						<.0001
Very hard	80 (10.8)	50 (15.6)	20 (6.9)	8 (17.4)	2 (2.5)	
Somewhat hard	235 (31.8)	128 (39.9)	65 (22.3)	25 (54.4)	17 (21.5)	
Not hard at all	423 (57.3)	143 (44.6)	207 (71.0)	13 (28.3)	60 (76.0)	
Menopausal status						<.0001
Surgical post	61 (7.6)	32 (9.1)	24 (7.7)	2 (3.6)	3 (3.5)	
Natural post	703 (87.7)	295 (84.1)	280 (90.3)	49 (87.5)	79 (92.9)	
Early/late peri	17 (2.1)	8 (2.3)	3 (1.0)	4 (7.2)	2 (2.4)	
Unknown^f^	21 (2.6)	16 (4.5)	3 (1.0)	1 (1.8)	1 (1.2)	
Years since FMP, mean (SD)	8.7 (3.2)	8.7 (3.1)	8.5 (3.0)	9.2 (4.4)	8.6 (3.6)	.33
Hormone users						.04
Current	51 (6.4)	14 (4.0)	28 (9.0)	2 (3.6)	7 (8.2)	
Past	288 (35.9)	112 (31.9)	136 (43.9)	12 (21.4)	28(32.9)	
Study Site						<.0001
Michigan	224 (27.9)	149 (42.5)	75 (24.2)	0	0	
MGH	123 (15.3)	80 (22.8)	43 (13.9)	0	0	
Chicago	102 (12.7)	65 (18.5)	37 (11.9)	0	0	
UC Davis	69 (8.6)	0	30 (9.7)	0	39 (45.9)	
UCLA	81 (10.1)	0	35 (11.3)	0	46 (54.1)	
New Jersey	71 (8.9)	0	15 (4.8)	56 (100)	0	
Pittsburgh	132 (16.5)	57 (16.2)	75 (24.2)	0	0	

*Note*: P‐values are calculated using chi‐Square (exact tests if appropriate) (categorical variables) and ANOVA or Kruskal‐Wallis tests (continuous variables). CVD, cardiovascular disease including stroke/transient ischemic attack, myocardial infarction, revascularization (coronary artery bypass grafting or percutaneous coronary intervention), or angina.

^a^ Participants with CAD/CVD may have diabetes and/LDL ≥190.

^b^ participants with DM but no history of cardiovascular disease (CVD) (she may have LDL≥190 but will be counted only in the diabetes group).

^c^ Participants without diagnosed CAD/CVD or diabetes but have had an LDL ≥190 at any visit from baseline to V12.

^d^ Hypertension definition: SBP ≥140 mmHg or DBP ≥90 mmHg or use of antihypertensive medications.

^e^ Family history of CAD definition: CAD (coronary artery disease) in first‐degree relative male ≤55 years or female ≤65 years.

^f^ Unknown menopausal status includes women taking hormonal therapy and/or have undergone a hysterectomy.

Abbreviations: ASCVD, atherosclerotic cardiovascular disease; BMI, body mass index; CAD, coronary artery disease; CVD, cardiovascular disease; FMP, final menstrual period.

In Table 3, we illustrate the rates of lipid‐lowering medication use by race/ethnicity among women who met the criteria for one of the four statin eligible groups. In general, rates of statin use were low across all women. Black women reported the lowest rate of statin use. Just over 55% of Asian women who met the criteria for statins were taking statins.

**TABLE 3:** Prevalence of lipid‐lowering medication use at Visit 12 among women eligible for statins

Lipid‐lowering medications^a^, n (%)	Total N = 802	Black N = 351	White N = 310	Hispanics N = 56	Asian N = 85	*P*‐value
Statins	367 (45.8)	143 (40.7)	152 (49.0)	25 (44.6)	47 (55.3)	.045
Fibrates	25 (3.1)	5 (1.4)	17 (5.5)	1 (1.8)	2 (2.4)	.024
Other^b^	39 (4.7)	10 (2.9)	18 (5.8)	6 (10.7)	5 (5.9)	.036

^a^ Participants can report being on multiple lipid‐lowering medications (33 women were on two lipid‐lowering medications, and one was on three lipid‐lowering medications.

^b^ Other includes Colesevelam (n = 5), Ezetimibe (n = 34).

Using the 2018 AHA/ACC cholesterol guidelines criteria for very high risk, we observed 78 women met this criterion. The majority (62.8%) of these women were Black, while 26.9% were White, 6.4% were Hispanic, and 3.9% were Asian. Rates of statin use were suboptimal in these women, with only 64.1% receiving statin therapy, including 43.8% of those with two or more ASCVD events and 69.4% of those with one major ASCVD event and multiple high‐risk conditions (Supplementary Table S1).

Among all statin eligible women, Black women had lower odds of reporting statin use (OR = 0.53, 95% CI: 0.36‐0.78) while Asian women had higher odds (OR = 1.85, 95% CI: 1.04‐3.29) compared to White women, after adjustment for age, BMI, education level, difficulty paying for basics, hormone use, menopausal status, HTN, current smoking, and family history of premature CAD (Table 4). HTN was also significantly associated with receiving statins (OR = 2.72, 95% CI: 1.81‐4.10).

**TABLE 4:** Factors related to statin use among women eligible for statins (N = 802)

	Unadjusted	Model 1	Model 2
Co‐variates	OR (95% CI)	OR (95% CI)	OR (95% CI)
Race/ethnicity			
White	Reference	Reference	Reference
Black	**0.72 (0.53‐0.97)**	**0.68 (0.47‐0.97)**	**0.53 (0.36‐0.78)**
Hispanic	0.84 (0.47‐1.49)	0.83 (0.40‐1.72)	0.68 (0.33‐1.44)
Asian	1.28 (0.79‐2.08)	**1.78 (1.02‐3.12)**	**1.85 (1.04‐3.29)**
Age		1.03 (0.97‐1.08)	1.01 (0.95‐1.07)
Body mass index		**1.03 (1.01‐1.05)**	
Educational level			
≤High school		0.86 (0.52‐1.42)	0.88 (0.53‐1.47)
>High school		0.65 (0.41‐1.02)	0.65 (0.41‐1.04)
Some college		0.71 (0.42‐1.20)	0.72 (0.42‐1.24)
Postcollege		Reference	
How hard to pay basics			
Very hard		1.49 (0.85‐2.62)	1.35 (0.77‐2.40)
Somewhat hard		1.05 (0.73‐1.51)	1.00 (0.69‐1.45)
Not hard at all		Reference	
Hormone user		1.16 (0.62‐2.16)	0.98 (0.52‐1.85)
Menopausal status			
Surgical post		1.69 (0.55‐5.21)	1.62 (0.52‐5.05)
Natural post		0.87 (0.31‐2.37)	0.83 (0.30‐2.27)
Early/late peri		0.69 (0.15‐3.23)	0.57 (0.12‐2.71)
Unknown due to hormone therapy or missing		Reference	
Hypertension			**2.72 (1.81–4.10)**

*Note*: Model 1 was adjusted for race/ethnicity, BMI, education, difficulty paying for basics, hormonal therapy use, menopausal status; Model 2 adjusted for variables in model 1, study site, current smoking status, family history of CAD (coronary artery disease) in first‐degree relative male ≤55 years or female ≤65 years and diagnosis of hypertension.

Bolded values were statistically significant, with a p value <0.05

Abbreviations: BMI, body mass index; CAD, coronary artery disease; CI, confidence interval; OR = odds ratio.

## 4 DISCUSSION

In this large, multiethnic cohort of midlife women, we observed the majority of women eligible for statins did not report statins use, with low rates across all racial/ethnic groups and clinical risk groups. Of note, Black women represented the group with the greatest percent of individuals eligible for statins; however, they were least likely to report taking statins.

### 4.1 Statin use in participants of SWAN with ASCVD is reduced

Statin therapy for adults with CVD has been supported by guidelines and trial evidence for several decades.^6^, ^17^, ^18^ Despite evidence‐based recommendations, several studies have observed statin use among women with CVD to be significantly lower than compared to men with CVD.^10^, ^13^, ^19^, ^20^, ^21^, ^22^ Although SWAN did not include men, the rates of statin use among women with CVD, were similar to several other studies.^23^, ^24^, ^25^, ^26^, ^27^, ^28^ Data from the medical expenditure panel survey (MEPS) demonstrated modestly higher rates of statin use among adults with ASCVD as of 2012 to 2013 compared to those of the SWAN women (58.1% vs 49.6%, respectively).^24^ However, the authors also noted a 19% lower rate of statin use among women compared to men. The MEPS data also demonstrated that only one‐third of adults with ASCVD were taking a high‐intensity statin, which has been observed in other studies.^24^, ^26^ Among Marketscan and Medical data, low rates of high‐intensity statin use among women with CVD have also been noted.^26^ It is likely that among SWAN women, rates of high‐intensity statin use would have been lower than the rate of reported statin use as well. Furthermore, Marketscan/Medicare data did not demonstrate an increase in statin use over the 2 years after the 2013 guideline publication.^26^ Unfortunately, data on statin dose was not available in SWAN. In a recently published study, which used Medicare and MarketScan data, statin rates were noted to be 52% for those with heart disease and 43% for those with cerebrovascular disease.^29^ These rates are minimally higher than we observed in SWAN; however, rates were not reported separately by gender. In a large meta‐analysis of 43 studies with over two million participants, rates of statin use were higher than we observed, with a pooled prevalence of statin use among 60% of women, which was significantly lower than the rates noted for men.^13^ It should also be noted that MEPS, Medicare, and MarketScan differ from SWAN as these data are collected through claims or surveys, which include healthcare providers and employers as data sources, as opposed to self‐report, which was used in SWAN. That said SWAN participants were asked to bring all current medications pill bottles with them to each visit for visual verification of reported medications.

Adults with elevated CVD risk are also recommended for statins therapy in prior guidelines.^6^, ^17^ Despite the evidence of benefit, just over 50% of SWAN women with diabetes reported taking a statin. This rate of statin use is similar data from MEPS.^24^ Among those with hyperlipidemia (LDL of 190 mg/dL), SWAN women reported similar rates of statin use as observed in MEPS, and the National Cardiovascular Data Registry Practice Innovation and Clinical Excellence Registry.^24^, ^30^

### 4.2 Black women are less likely to use statins

Prior studies have observed lower rates of evidence‐based therapies in CVD prevention among minority populations.^20^, ^23^, ^27^, ^31^ Salami et al., using MEPs data, noted Blacks were 35% less likely to receive statins compared to non‐minorities.^24^ Similar rates were observed for Hispanics. Data from the National Ambulatory Medical Care Survey and the National Hospital Ambulatory Medical Care Survey also support the underuse of statins among women and minorities.^25^ The Patients and provider assessment of lipid management (PALM) registry also demonstrated lower rates of guideline‐recommended statin use among Black adults as compared to Whites.^32^ The PALM investigators reported that Black participants were more likely to report beliefs that statins were not safe or effective compared to White participants. Other studies have noted that women are more likely to stop statins as compared to men, although results were not reported separately by race and gender.^33^, ^34^

### 4.3 SES factors are associated with the use of statins

SES factors may explain differences in statin rates among SWAN women. Lower educational attainment and more difficulty paying for basics were more prevalent among statin eligible Black and Hispanic women compared to White or Asian women. However, in multivariable models, these factors did not appear to be associated with receipt of statins. Data from Finland suggests lower SES was associated with lower statin adherence among men but not women.^35^ However, Finland has universal health coverage, which limits generalizability to the United States. In the MEPS data, investigators observed no difference in statin use for when the family income was less than 100% of the federal poverty Level; however, being uninsured was associated with lower rates of statin use compared to having public or private insurance.^24^

### 4.4 Study limitations

Our study has several limitations. First, we did not have information on the dose of statins for the majority of women. Therefore, we were unable to determine if women were on recommended doses. We also do not have information on the provider‐measured lipid profiles, which may have triggered a statin prescription; however, this would not be relevant for recommendations to use statin in women with CVD or diabetes. Second, SWAN data was collected shortly before the publication of the 2013 guidelines, which outlined specific definitions for the four groups recommended for statin therapy. However, three of these groups (those with CVD, diabetes, or elevated LDL cholesterol) would have been recommended for statins therapy based on earlier guidelines^6^; thus majority of SWAN women met these earlier recommendations. Among women who did not meet the definitions for one of the four benefit groups, some women reported being on statins. These women may have had an ASCVD risk score, which was higher in the past, but subsequent statin use reduced their risk score. This limitation would not influence the observed rates of statin use for women with CVD, diabetes or a history of an elevated LDL of 190 mg/dL or greater, but could mean the rate of statin use among women with an ASCVD risk score of 7.5% was higher than we observed. We also could not ascertain if racial differences in statin use reflect differences in adherence or provider recommendations. Lastly, ASCVD events were self‐reported, which could lead to misclassification.

## 5 CONCLUSION

This study suggests that women who are eligible for statin therapy are commonly not taking statins, and in particular Black women report low rates of statin use. Strategies to increase statin use among women at risk for future CVD, including minority women, should be considered.

## Supporting information




**Table S1** Characteristics of Women at Very High Risk for Future ASCVD Events
