# Supplementary material for: Is race or ethnicity associated with under‐utilization of statins among women in the United States: The study of women's health across the nation
Source: Clin Cardiol. 2020 Aug 30;43(12):1388–97. doi: 10.1002/clc.23448 (PMC7724205; doi:10.1002/clc.23448)
Supplement: Supplementary file 1 — Table S1 Characteristics of Women at Very High Risk for Future ASCVD Events [file CLC-43-1388-s001.docx]

**Supplementary Table. Characteristics of Women at Very High Risk for Future ASCVD Events**

| **Characteristic, n (%)** | **Total**  **N= 78** | **Two or More ASCVD Events^†^**  **N= 16** | **One Major ASCVD Event + Multiple High-Risk Conditions^††^**  **N= 62** | **P-Value** |
| --- | --- | --- | --- | --- |
| Age, years, mean (SD) | 60.8 (3.0) | 60.9 (3.7) | 60.8 (2.9) | 0.91 |
| **Race/Ethnicity** |  |  |  | 0.06 |
| White | 21 (26.9) | 2 (12.5) | 19 (30.7) |  |
| Black | 49 (62.8) | 10 (62.5) | 39 (62.9) |  |
| Hispanic | 5 (6.4) | 2 (12.5) | 3 (4.8) ‚ |  |
| Asian | 3 (3.9) | 2 (12.5) | 1 (1.6) |  |
| **Education** |  |  |  | 0.94 |
| ≤High School | 26 (33.3) | 6 (37.5) | 20 (32.3) |  |
| >High School | 38 (48.7) | 8 (50.0) | 30 (48.4) |  |
| College | 9 (11.5) | 1 (6.3) | 8 (12.9) |  |
| Post | 5 (6.4) | 1 (6.3) | 4 (6.5) |  |
| **Income** |  |  |  | 0.31 |
| Less than $20,000 | 27 (42.9) | 6 (40.0) | 21 (43.8) |  |
| $ 20,000-35,000 | 14 (22.2) | 4 (26.7) | 10 (20.8) |  |
| $ 35,000-50,000 | 4 (6.4) | 2 (13.3) | 2 (4.2) |  |
| $ 50,000-75,000 | 10 (15.9) | 3 (20.0) | 7 (14.6) |  |
| $ 75,000 or more | 8 (12.7) | 0 | 8 (16.7) |  |
| **How hard to pay basics** |  |  |  | 0.25 |
| Very hard | 16 (23.5) | 2 (14.3) | 14 (25.9) |  |
| Somewhat hard | 29 (42.7) | 9 (64.3) | 20 (37.0) |  |
| Not hard at all | 23 (33.8) | 3 (21.4) | 20 (37.0) |  |
| Hypertension* | 70 (92.1) | 13 (86.7) | 57 (93.4) | 0.34 |
| Family history of CAD^#^ | 56 (76.7) | 10 (71.4) | 46 (78.0) | 0.72 |
| Current Smoker | 29 (39.2) | 7 (43.8) | 22 (37.9) | 0.78 |
| BMI, (kg/m^2^), mean (SD) | 34.2 (7.2) | 32.9 (5.1) | 34.6 (7.7) | 0.46 |
| Waist circumference, cm, mean (SD) | 104.6 (14.2) | 104.5 (11.3) | 104.6 (15.0) | 0.98 |
| Waist hip ratio, mean (SD) | 0.9 (0.1) | 0.9 (0.1) | 0.9 (0.1) | 1 |
| Systolic Blood Pressure (mmHg), mean (SD) | 131.0 (18.0) | 137.4 (15.2) | 129.2 (18.4) | 0.13 |
| Diastolic Blood Pressure (mmHg), mean (SD) | 75.6 (10.4) | 75.9 (14.1) | 75.5 (9.3) | 0.90 |
| Total Cholesterol (mg/dl), mean (SD) | 183.3 (47.1) | 191.3 (39.3) | 181.1 (49.1) | 0.40 |
| High Density Lipoprotein (mg/dl), mean (SD) | 46.9 (14.3) | 51.4 (24.3) | 45.7 (10.1) | 0.86 |
| Low Density Lipoprotein (mg/dl), mean (SD) | 107.2 (38.0) | 112.7 (36.6) | 105.6 (38.6) | 0.47 |
| Triglycerides (mg/dl), mean (SD) | 150.5 (76.1) | 135.4 (49.5) | 154.7 (81.8) | 0.68 |
| Statin Therapy | 50 (64.1) | 7 (43.8) | 43 (69.4) | 0.08 |
| Ezetimibe Therapy | 7 (9.0) | 2 (12.5) | 5 (8.1) | 0.63 |

* Hypertension definition: SBP ≥140 mmHg or DBP ≥ 90 mmHg or use of antihypertensive medications.

^#^ Family history of CAD definition: CAD (coronary artery disease) in first-degree relative male ≤55 years or female ≤65 years.

^†^ ASCVD Events includes Myocardial Infarction, Ischemic Stroke, and PAD/Lower Extremity Procedure

^††^ High-Risk Conditions includes age ≥ 65 years of age, LDL ≥ 190 mg/dL at any visit, history of revascularization (CABG or PCI without concurrent myocardial infarction), History of congestive heart failure, diabetes mellitus, hypertension, and current smoking at visit 12

SD = standard deviation; BMI = body mass index;
